# Choriocarcinoma in the Cervix: Case Report of a Challenging Differential Diagnosis

**DOI:** 10.1155/crog/3427582

**Published:** 2026-03-08

**Authors:** Kristin Pfister, Rok Satler, Dieter Erdin, Andreas Müller, Gesine Meili, Peter Bode

**Affiliations:** ^1^ Institute of Pathology, Cantonal Hospital Winterthur, Winterthur, Switzerland, ksw.ch; ^2^ Clinic of Gynaecology, Cantonal Hospital Winterthur, Winterthur, Switzerland, ksw.ch; ^3^ Clinic of Oncology, Cantonal Hospital Winterthur, Winterthur, Switzerland, ksw.ch

## Abstract

**Background:**

Primary cervical choriocarcinoma in abortion curettage tissue is extremely rare and represents a diagnostic challenge. It is a serious condition that requires prompt and targeted therapeutic management. This case report describes the diagnostic evaluation, taking into account relevant differential diagnoses and highlighting the importance of a comprehensive diagnostic work‐up to resolve these complex cases.

**Case report:**

A 43‐year‐old woman presented with recurrent vaginal bleeding, an elevated *β*‐HCG of 1165 mU/mL, and sonographic suspicion of an incomplete abortion with an ectopic pregnancy in the cervix. Dilation and curettage were performed. Histological analysis raised the suspicion of a gestational choriocarcinoma of the cervix. In contrast, an external specialist referral suggested a somatic carcinoma with germ cell differentiation due to an abnormal p53 pattern, which subsequently led to a change in the management. The patient underwent a total hysterectomy and bilateral salpingo‐oophorectomy together with bilateral iliac lymphadenectomy. In the final molecular workup, nonmaternal DNA was identified in the tumor tissue. A *TP53* mutation could not be detected. In summary, these findings confirmed a gestational choriocarcinoma of the cervix. Follow‐up 28 months after completion of chemotherapy revealed no evidence of recurrence.

**Conclusion:**

Cervical gestational choriocarcinoma requires thorough molecular investigation, as accurate diagnosis is pivotal for optimal management.

**Summary**

• In the case of suspicious findings in the cervix and elevated *β*‐HCG levels, cervical choriocarcinoma should be considered.
• Distinguishing gestational from nongestational choriocarcinoma is often extremely challenging, if not impossible, by histology alone. Therefore, molecular analyses are essential for accurate diagnosis.

## 1. Introduction

Choriocarcinomas are rare malignant trophoblastic neoplasms that can arise in association with pregnancy or in a nongestational setting, with the gestational form being more common [1]. They typically present in the uterine corpus. Ectopic primary cervical gestational choriocarcinoma is extremely rare and therefore diagnostically challenging [1, 2]. The differential diagnosis includes a nongestational choriocarcinoma. They can arise from germ cell origin—pure or as part of a mixed germ cell tumor—or from somatic origin [1, 3]. Since the diagnosis cannot be established by morphology alone, molecular analyses are required.

## 2. Case

A 43‐year‐old woman (G3 C1A) presented with recurrent vaginal bleeding for 2 months and lower abdominal pain. She had a positive pregnancy test with a *β*‐HCG of 1165 mU/mL. Her medical history included a cesarean section 7 years prior and a missed abortion 18 years prior. Transvaginal ultrasound revealed an empty uterine cavity and a cervical mass measuring 42 × 21 mm. The mass appeared hypoechogenic, hypervascularized, and poorly demarcated (Figure 1a). Due to persistent bleeding secondary to a suspected incomplete abortion, a dilation and curettage was performed with laparoscopic backup in case of a cervical ectopic pregnancy. Histological analysis of the largely hemorrhagic, fragmented tissue showed a biphasic tumor composed of pleomorphic mononuclear cells and interspersed multinucleated tumor cells with extensive areas of necrosis (Figure 1b,c). The diagnosis of cervical gestational choriocarcinoma was confirmed using immunohistochemistry (Figure 1d,e). In addition, molecular workup was initiated. Furthermore, a second external histopathological evaluation was requested due to the rare entity. A staging PET‐CT and cranial MRI showed no evidence of distant metastases. The postoperative *β*‐HCG level dropped to 4 mU/mL. Per the FIGO and WHO Prognostic Scoring System, the case was classified as FIGO Stage I:4. The multidisciplinary tumor board recommended single‐agent chemotherapy with methotrexate.

Figure 1Ultrasound, HE, morphology, and immunohistochemical profile of the biphasic tumor in cervical abortion curettage tissue. (a) Transvaginal ultrasound with cervical tumor mass (arrow). (b) Overview of cell‐dense tumor (HE, ×5). (c) Medium‐sized pleomorphic mononuclear cells and scattered multinuclear giant cells (arrow, HE, ×40). (d) Immunohistochemical positivity of giant cells for *β*‐HCG (arrow, *β*‐HCG, ×40). (e) Positive immunohistochemical nuclear staining with SALL4 (SALL4, ×10). (f) p53 immunohistochemistry: heterogeneous (wild‐type) staining pattern with p53 (p53, ×20).

**Figure:**
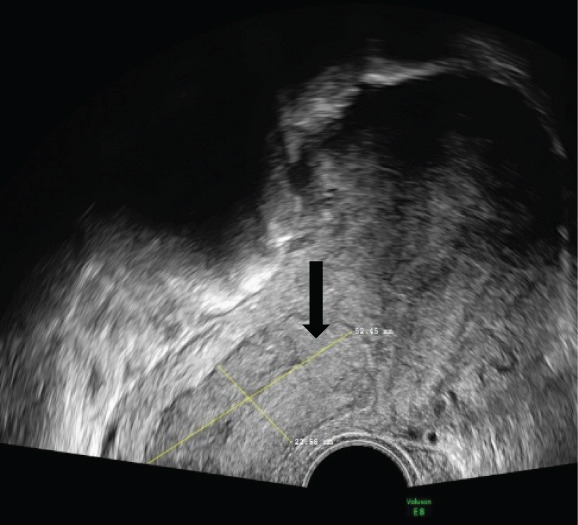
(a)

**Figure:**
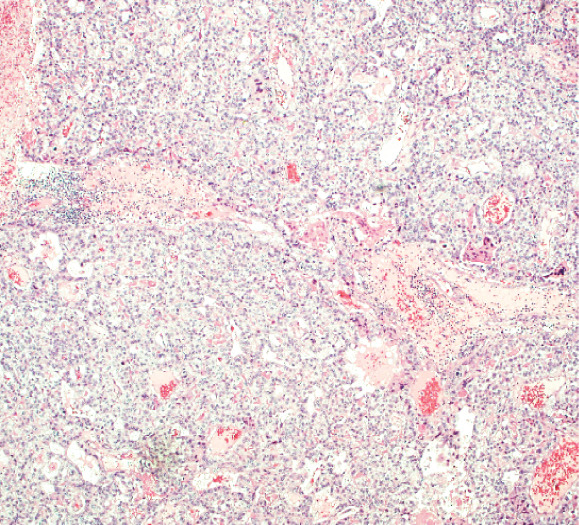
(b)

**Figure:**
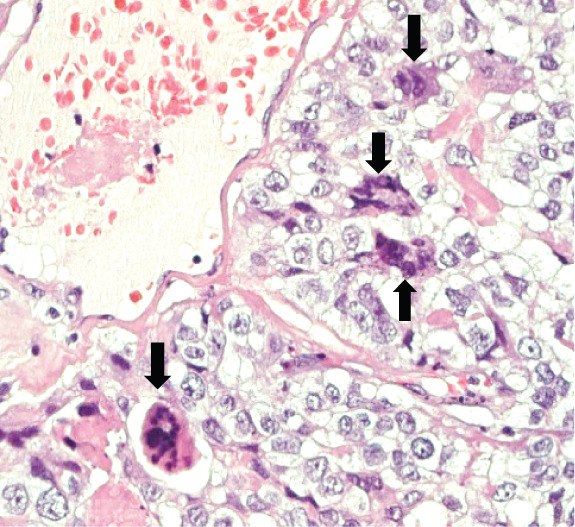
(c)

**Figure:**
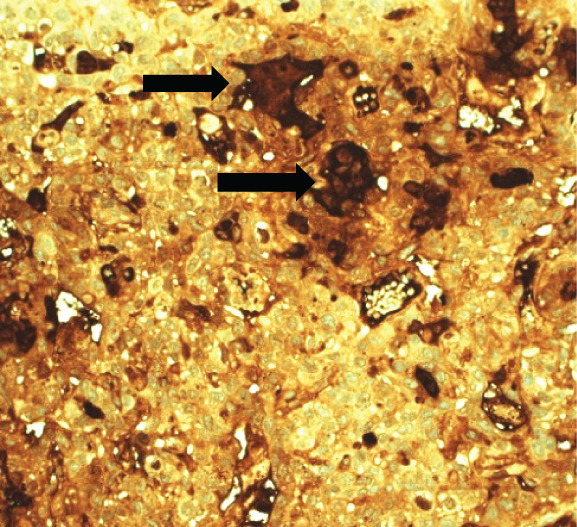
(d)

**Figure:**
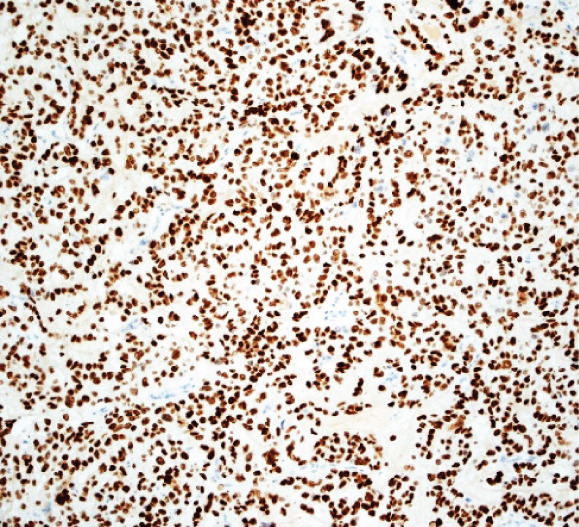
(e)

**Figure:**
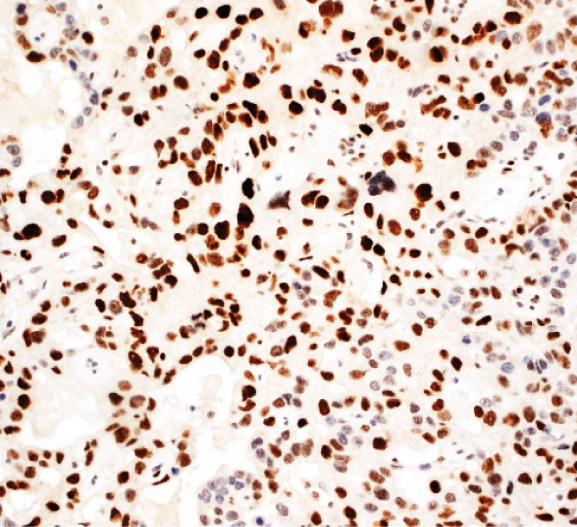
(f)

Surprisingly, the external pathologists providing the second opinion favored a somatic carcinoma with germ cell differentiation, based on an abnormal p53 staining pattern. Therapeutic management was subsequently escalated, and the patient underwent a total hysterectomy with bilateral salpingo‐oophorectomy and resection of the bilateral iliac lymph nodes. No residual tumor was detected in the uterus, and no lymph node metastases were identified.

Following completion of therapy, the results of the molecular analyses finally demonstrated nonmaternal DNA in the tumor tissue. Furthermore, a *TP53* mutation could not be confirmed at either the molecular or immunohistochemical level (molecular results summarized in Table 1, Figure 1f). These findings confirmed the initial diagnosis of cervical gestational choriocarcinoma. Subsequent clinical *β*‐HCG follow‐up testing has been unremarkable. The patient remains disease‐free 28 months after completion of treatment.

**Table 1:** Summary of relevant molecular alterations performed.

	Findings	Interpretation
Thermo Fisher OncoScan CNV Assay	‐ Heterozygous loss involving the TP53 region on chromosome 17‐ Genomic instability‐ Isochromosome i12p or 12p gains not detected	Presumptive diagnosis of a carcinoma of somatic origin
FoundationOne	‐ Detection of nonmaternal DNA‐ TP53 mutation not detected‐ No alterations on chromosome 12 or any other specific genetic changes	Somatic carcinoma and primary germ cell tumor can be excludedDetection of nonmaternal DNA ultimately confirms a gestational choriocarcinoma
Microsatellite analysis	Detection of nonmaternal DNA	The diagnosis of gestational choriocarcinoma was repeatedly confirmed

## 3. Discussion

We report the case of a woman who initially presented with suspected incomplete abortion. Histology showed a distinctive biphasic morphology composed of mononuclear cytotrophoblastic and multinucleated syncytiotrophoblastic cells—the typical pattern of a choriocarcinoma (Figure 1). However, a thorough genetic investigation is required to differentiate gestational choriocarcinoma from nongestational forms [1, 4, 5]. These nongestational forms originate from germ cells (either as pure choriocarcinoma or as mixed germ cell tumor with a choriocarcinomatous component) or from somatic cells induced to pluripotency (somatic high‐grade carcinoma exhibiting germ cell differentiation) [1, 3]. This distinction is crucial, as prognosis and therapeutic approaches differ substantially (type of chemotherapy and extent of surgery).

Cervical gestational choriocarcinomas are extremely rare. They can mimic a cervical ectopic pregnancy, with nonspecific vaginal bleeding being the most common presenting symptom [2, 4, 5]. Saito et al. [6] defined diagnostic criteria for cervical choriocarcinoma, including

1. Absence of evidence for a primary uterine corpus choriocarcinoma,
2. Histopathological confirmation, and
3. Exclusion of extrauterine choriocarcinoma coexisting with a hydatiform mole or normal intrauterine pregnancy accompanied by intramural choriocarcinoma.

Gestational choriocarcinomas typically arise in the uterine corpus, and in over 50% of cases, they are preceded by a complete hydatidiform mole [1, 4, 7]. Histomorphologically, these tumors are characterized by infiltrative and destructive growth patterns [7]. The morphological resemblance to cervical squamous cell carcinomas can be problematic, making immunohistochemical markers such as *β*‐HCG and SALL4 essential within a broader panel [7–11]. At the molecular level, detection of nonmaternal DNA of paternal origin confirms the diagnosis. It also allows the distinction between molar (purely androgenetic) and biparental gestational types [1, 12–14].

Uterine gestational choriocarcinoma can often be managed with chemotherapy alone, whereas cervical gestational choriocarcinomas frequently require primary hysterectomy [2]. Alternatively, uterine artery embolization may be considered in cases of massive hemorrhage when fertility preservation is desired, allowing conservative management with chemotherapy [15, 16]. Nevertheless, recurrence rates appear to be higher for cervical gestational choriocarcinoma compared to those in other anatomical locations—particularly when hysterectomy is not performed—although conclusions are limited due to sparse data [2]. Chemotherapy remains essential in the treatment of gestational trophoblastic disease, with single‐agent regimens recommended for low‐risk tumors (FIGO score < 7) and multiagent therapy for high‐risk choriocarcinomas (FIGO score ≥ 7) [17].

Nongestational choriocarcinoma is a germ cell tumor arising from late primordial germ cells or gonocytes. It affects children and young adults, demonstrates elevated *β*‐HCG, and accounts for < 1% of all ovarian neoplasms [7]. Extragonadal germ cell tumors may arise in periadnexal or pelvic locations [1, 5, 13]. Primary true germ cell tumors of the cervix—such as mature teratomas, dermoid cysts, and yolk sac tumors—may occur in younger patients but are exceptionally rare [18]. In contrast to gestational choriocarcinoma, DNA genotyping reveals an allelic pattern identical to the patient’s own DNA [13]. Genetic abnormalities involving chromosome 12, particularly isochromosome 12p, are frequently observed [19]. Nongestational choriocarcinomas exhibit a more aggressive clinical course, with lymphatic and intraperitoneal spread and poor response to chemotherapy compared to their gestational counterparts [1, 5, 18, 20]. Commonly, the BEP (bleomycin, etoposide, and cisplatin) regimen is used in analogy to the treatment of malignant germ cell tumors.

The third differential diagnosis is somatic carcinoma with germ cell differentiation [21, 22]. These tumors contain Müllerian epithelial components together with germ cell tumor elements or trophoblastic cells [3]. They predominantly occur in peri‐ or postmenopausal women and often show yolk sac differentiation [23]. The non‐Müllerian component usually dominates and displays both characteristic morphology and immunohistochemical profiles [3]. Some case reports describe choriocarcinomatous differentiation [8, 24, 25]. Several hypotheses exist regarding the origin of these tumors, including metaplastic transformation of cervical epithelium or secondary cervical involvement representing metastasis from a completely regressed uterine primary [26]. Sequencing studies show distinct genetic profiles compared to gestational choriocarcinoma [27]. Shared mutation profiles (e.g., *TP53*, *ARID1A*, and *PIK3CA*) in both Müllerian and non‐Müllerian components suggest a common clonal origin [28]. The absence of isochromosome 12p and the presence of multiple copy‐number variants point toward underlying genomic instability [3, 27]. Although these tumors may resemble germ cell tumors morphologically, their behavior is more in line with high‐grade Müllerian‐derived somatic carcinomas, exhibiting poor chemotherapy response and early recurrence. The chemotherapy regimen is usually a polychemotherapy regimen directed at the specific somatic histology rather than BEP [23, 25, 29–31]. Therefore, radical surgery is often required [8, 24, 32].

In our case, molecular analysis was crucial. The absence of 12p alterations excluded a primary germ cell tumor. Furthermore, the initially suspected that the *TP53* mutation could not be confirmed, and no other pathogenic genetic alterations were detected; therefore, somatic carcinoma could be ruled out. Finally, the detection of nonmaternal DNA ultimately confirmed the diagnosis of a gestational choriocarcinoma (molecular tests and results summarized in Table 1).

## 4. Conclusion

Cervical gestational choriocarcinoma is very rare, making the diagnosis challenging. In premenopausal patients with vaginal bleeding and elevated *β*‐HCG levels, prompt histological and molecular workup is crucial to ensure accurate diagnosis and appropriate management. In cases of massive bleeding, hysterectomy or arterial embolization may be required. Depending on the risk stratification of the underlying tumor, single‐agent or multiagent chemotherapy is recommended.

## Author Contributions

Kristin Pfister and Rok Satler contributed equally to this work.

## Funding

No funding was received for this manuscript.

## Consent

Written informed consent for publication of this case report was obtained from the patient.

## Conflicts of Interest

The authors declare no conflicts of interest.

## Data Availability

The data that support the findings of this study are available from the corresponding author upon reasonable request.

## References

[1] Savage J. , Adams E. , Veras E. , Murphy K. M. , and Ronnett B. M. , Choriocarcinoma in Women: Analysis of a Case Series With Genotyping, American journal of surgical pathology. (2017) 41, no. 12, 1593–1606, 10.1097/PAS.0000000000000937, 2-s2.0-85034740708, 28877059. 28877059

[2] Wang X. , Yang J. , Wan X. , Feng F. , Zhao J. , Ren T. , and Xiang Y. , Identification and Treatment of Primary Cervical Gestational Trophoblastic Neoplasia: A Retrospective Study of 13 Patients and Literature Review, Orphanet Journal of Rare Diseases. (2021) 16, no. 1, 10.1186/s13023-021-02111-w, 34794477. PMC8600730 34794477

[3] Acosta A. M. , Sholl L. M. , Cin P. D. , Howitt B. E. , Otis C. N. , and Nucci M. R. , Malignant Tumours of the Uterus and Ovaries With Mullerian and Germ Cell or Trophoblastic Components Have a Somatic Origin and Are Characterised by Genomic Instability, Histopathology. (2020) 77, no. 5, 788–797, 10.1111/his.14188, 32558949. 32558949

[4] Hoffner L. and Surti U. , The Genetics of Gestational Trophoblastic Disease: A Rare Complication of Pregnancy, Cancer Genet. (2012) 205, no. 3, 63–77, 10.1016/j.cancergen.2012.01.004, 2-s2.0-84860230336. 22469506

[5] World Health Organization , Classification of Tumours Editorial Board, Female Genital Tumours. (2020) 5th edition, WHO.

[6] Saito M. , Azuma T. , and Nakamura K. , On Ectopic Choriocarcinoma, World Journal of Obstetrics and Gynecology. (1963) 17, 459–484.

[7] Hui P. , Gestational Trophoblastic Tumors: A Timely Review of Diagnostic Pathology, Archives of Pathology & Laboratory Medicine. (2019) 143, no. 1, 65–74, 10.5858/arpa.2018-0234-RA, 2-s2.0-85059777742, 30407075. 30407075

[8] Baykal C. , Tulunay G. , Bülbül D. , Boran N. , and Köse M. F. , Primary Choriocarcinoma of the Uterine Cervix in a Postmenopausal Patient: A Case Report, Gynecologic Oncology. (2003) 90, no. 3, 667–669, 10.1016/s0090-8258(03)00369-x, 2-s2.0-0141974964, 13678743. 13678743

[9] Kalhor N. , Ramirez P. T. , Deavers M. T. , Malpica A. , and Silva E. G. , Immunohistochemical Studies of Trophoblastic Tumors, American Journal of Surgical Pathology. (2009) 33, no. 4, 633–638, 10.1097/PAS.0b013e318191f2eb, 2-s2.0-63849125311. 19145204

[10] Stichelbout M. , Devisme L. , Franquet-Ansart H. , Massardier J. , Vinatier D. , Renaud F. , and Kerdraon O. , SALL4 Expression in Gestational Trophoblastic Tumors: A Useful Tool to Distinguish Choriocarcinoma From Placental Site Trophoblastic Tumor and Epithelioid Trophoblastic Tumor, Human Pathology. (2016) 54, 121–126, 10.1016/j.humpath.2016.03.012, 2-s2.0-84971220915, 27068524. 27068524

[11] Shi J. and Chen P. , Diagnostic Pitfall in Primary Cervical Gestational Choriocarcinoma: A Case Report, Frontiers in Oncolog. (2025) 15, 1608856, 10.3389/fonc.2025.1608856, 40936718. PMC12420282 40936718

[12] Kanehira K. , Starostik P. , Kasznica J. , and Khoury T. , Primary Intraplacental Gestational Choriocarcinoma: Histologic and Genetic Analyses, International Journal of Gynecological Pathology. (2013) 32, no. 1, 71–75, 10.1097/PGP.0b013e3182566552, 2-s2.0-84871643583, 23202784. 23202784

[13] Zhang X. , Yan K. , Chen J. , and Xie X. , Using Short Tandem Repeat Analysis for Choriocarcinoma Diagnosis: A Case Series, Diagnostic Pathology. (2019) 14, no. 1, 10.1186/s13000-019-0866-5, 2-s2.0-85070990095, 31421690. PMC6698345 31421690

[14] Jiao L. , Ghorani E. , Sebire N. J. , and Seckl M. J. , Intraplacental Choriocarcinoma: Systematic Review and Management Guidance, Gynecologic Oncology. (2016) 141, no. 3, 624–631, 10.1016/j.ygyno.2016.03.026, 2-s2.0-84962424911, 27020699. 27020699

[15] Frati A. , Ducarme G. , Wernet A. , Chuttur A. , Vilgrain V. , and Luton D. , Uterine Artery Embolization as Treatment for Life-Threatening Haemorrhage From a Cervical Choriocarcinoma: A Case Report, European Journal of Obstetrics and Gynecology and Reproductive Biology. (2008) 141, no. 1, 87–88, 10.1016/j.ejogrb.2008.05.008, 2-s2.0-54849418875, 18649986. 18649986

[16] Chandacham A. , Kietpeerakool C. , Khunamornpong S. , Suprasert P. , Srisomboon J. , Charoenkwan K. , Phongnarisorn C. , Cheewakraingkrai C. , Siriaree S. , and Tantipalakorn C. , Successfully Conservative Treatment of Large Cervical Choriocarcinoma With Profuse Vaginal Bleeding, Medical journal of the Medical Association of Thailand. (2009) 92, no. 1, 120–123, 19260253. 19260253

[17] Kong Y. , Zong L. , Cheng H. , Jiang F. , Wan X. , Feng F. , Ren T. , Zhao J. , Yang J. , and Xiang Y. , Management and Risk Factors of Recurrent Gestational Trophoblastic Neoplasia: An Update From 2004 to 2017, Cancer Medicine. (2020) 9, no. 7, 2590–2599, 10.1002/cam4.2901, 32022487. 32022487 PMC7131839

[18] Nasioudis D. , Chapman-Davis E. , Frey M. K. , Caputo T. A. , and Holcomb K. , Management and Prognosis of Ovarian Yolk Sac Tumors; An Analysis of the National Cancer Data Base, Gynecologic Oncology. (2017) 147, no. 2, 296–301, 10.1016/j.ygyno.2017.08.013, 2-s2.0-85028075885. 28803748

[19] Poulos C. , Cheng L. , Zhang S. , Gersell D. J. , and Ulbright T. M. , Analysis of Ovarian Teratomas for Isochromosome 12p: Evidence Supporting a Dual Histogenetic Pathway for Teratomatous Elements, Modern Pathology. (2006) 19, no. 6, 766–771, 10.1038/modpathol.3800596, 2-s2.0-33646873910, 16547466. 16547466

[20] Ahn S. H. , Roh H. J. , Cho H. J. , You S. G. , Lee S. H. , and Kwon Y. S. , Pure Non-Gestational Choriocarcinoma Arising in the Ovary, European Journal of Gynaecological Oncology. (2016) 37, no. 4, 549–553, 29894083. 29894083

[21] Oosterhuis J. W. and Looijenga L. H. , Testicular Germ-Cell Tumours in a Broader Perspective, Nature Reviews Cancer. (2005) 5, no. 3, 210–222, 10.1038/nrc1568, 2-s2.0-14644396631, 15738984. 15738984

[22] Oosterhuis J. W. and Looijenga L. H. J. , Human Germ Cell Tumours From a Developmental Perspective, Nature Reviews Cancer. (2019) 19, no. 9, 522–537, 10.1038/s41568-019-0178-9, 2-s2.0-85071036122. 31413324

[23] Ravishankar S. , Malpica A. , Ramalingam P. , and Euscher E. D. , Yolk Sac Tumor in Extragonadal Pelvic Sites: Still a Diagnostic Challenge, American Journal of Surgical Pathology. (2017) 41, no. 1, 1–11, 10.1097/PAS.0000000000000722, 2-s2.0-84987923136. 27631522

[24] Maestá I. , Michelin O. C. , Traiman P. , Hokama P. , and Rudge M. V. , Primary Non-Gestational Choriocarcinoma of the Uterine Cervix: A Case Report, Gynecologic Oncology. (2005) 98, no. 1, 146–150, 10.1016/j.ygyno.2005.03.045, 2-s2.0-20444447441, 15925400. 15925400

[25] Hwang D. W. , Song H. S. , Choi Y. Y. , Kim H. S. , Kim Y. A. , and Chun K. C. , Primary Non-Gestational Choriocarcinoma of the Uterine Cervix With Metaplastic Transformation From Adenocarcinoma: A Case Report, Journal of Obstetrics and Gynaecology. (2018) 38, no. 2, 289–290, 10.1080/01443615.2017.1336756, 2-s2.0-85028548322, 28830246. 28830246

[26] Longo R. , Battaglia F. , Gattuso D. , De Sio L. , Sarmiento R. , Amici S. , and Gasparini G. , Primary Nongestational Choriocarcinoma of the Uterine Cervix, Journal of Clinical Oncology. (2011) 29, no. 11, e301–e302, 10.1200/JCO.2010.33.2361, 2-s2.0-79955039882, 21245425. 21245425

[27] Xing D. , Zheng G. , Pallavajjala A. , Schoolmeester J. K. , Liu Y. , Haley L. , Hu Y. , Liu L. , Logan L. , Lin Y. , Pearce K. E. , Sattler C. A. , Tsai Y. C. , Vang R. , Hung C. F. , Wu T. C. , and Ronnett B. M. , Lineage-Specific Alterations in Gynecologic Neoplasms With Choriocarcinomatous Differentiation: Implications for Origin and Therapeutics, Clinical Cancer Research. (2019) 25, no. 14, 4516–4529, 10.1158/1078-0432.CCR-18-4278, 2-s2.0-85069056946, 31010836. 31010836

[28] Skala S. L. , Liu C. J. , Udager A. M. , and Sciallis A. P. , Molecular Characterization of Uterine and Ovarian Tumors With Mixed Epithelial and Germ Cell Features Confirms Frequent Somatic Derivation, Modern Pathology. (2020) 33, no. 10, 1989–2000, 10.1038/s41379-020-0548-6. 32404953

[29] Pavelka J. C. , Bryant D. A. , and Vaccarello L. , Adenocarcinoma of the Uterine Cervix With Choriocarcinomatous Metastasis, Gynecologic Oncology. (2006) 101, no. 2, 346–348, 10.1016/j.ygyno.2005.11.021, 2-s2.0-33646388953, 16430947. 16430947

[30] Wang Y. , Yang J. , Yu M. , Cao D. , Zhang Y. , Zong X. , and Shen K. , Ovarian Yolk Sac Tumor in Postmenopausal Females: A Case Series and a Literature Review, Medicine (Baltimore). (2018) 97, no. 33, e11838, 10.1097/MD.0000000000011838, 2-s2.0-85052175688, 30113473. 30113473 PMC6112915

[31] Umemori Y. , Hiraki A. , Aoe K. , Murakami T. , Maeda T. , Matsuda E. , and Takeyama H. , Primary Choriocarcinoma of the Lung, Anticancer Research. (2004) 24, no. 3b, 1905–1910, 15274374. 15274374

[32] Chumworathayi B. and Kleebkaow P. , Primary Non-Gestational Uterine Cervical Choriocarcinoma With Metaplastic Transformation From Squamous Cells, Asian Pacific Journal of Cancer Prevention. (2007) 8, no. 4, 642–644, 18260746. 18260746

